# Accuracy and Technical Predictability of Computer Guided Bone Harvesting from the Mandible: A Cone-Beam CT Analysis in 22 Consecutive Patients

**DOI:** 10.3390/jfb13040292

**Published:** 2022-12-10

**Authors:** Luca De Stavola, Alessandro Cristoforetti, Andrea Fincato, Giandomenico Nollo, Paolo Ghensi, Anna Cantarutti, Francesco Tessarolo

**Affiliations:** 1 Department of Neurosciences, School of Dentistry, University of Padua, 35121 Padua, Italy; 2 Department of Industrial Engineering, University of Trento, 38123 Trento, Italy; 3 Department CIBIO, University of Trento, 38123 Trento, Italy; 4 Department of Statistics and Quantitative Methods, University of Milano-Bicocca, 20126 Milan, Italy; 5 Healthcare Research and Innovation Program (IRCS-FBK-PAT), Bruno Kessler Foundation, 38123 Trento, Italy

**Keywords:** bone harvesting, computer assisted surgery, accuracy, predictability, cone beam computed tomography

## Abstract

This study assesses the accuracy and technical predictability of a computer-guided procedure for harvesting bone from the external oblique ridge using a patient-specific cutting guide. Twenty-two patients needing bone augmentation for implant placement were subjected to mandibular osteotomy employing a case-specific stereolithographic surgical guide generated through computer aided design. Differences between planned and real cut planes were measured comparing pre- and post-operative Cone Beam Computed Tomography images of the donor site according to six validated angular and displacement indexes. Accuracy and technical predictability were assessed for 119 osteotomy planes over the study population. Three different guide fitting approaches were compared. An average root-mean-square discrepancy of 0.52 (0.30–0.97) mm was detected. The accuracy of apical and medial planes was higher than the mesial and distal planes due to occasional antero-posterior guide shift. Fitting the guide with an extra reference point on the closest tooth performed better than using only the bone surface, with two indexes significantly lower and less disperse. The study showed that the surgical plan was actualized with a 1 mm safety margin, allowing effective nerve preservation and reducing technical variability. When possible, surgical guide design should allow fitting on the closest tooth based on both radiological and/or intra-oral scan data.

## 1. Introduction

During prosthodontic restorations, optimal implant placement in the presence of alveolar crest atrophy or defects is strongly dependent on bone augmentation procedures [1,2]. Autogenous bone is the most predictable material to support new bone formation, allowing for a higher bone survival rate and implant success [3,4,5,6,7]. Harvesting in the intraoral donor sites, such as in the retromolar region of the mandibular ramus, is effective and safe for treating up to medium-size alveolar defects [8], and many freehand bone harvesting approaches have been described in the literature, making use of different tools, such as burs, diamond discs, or piezo surgery [9,10,11]. Avoiding mandibular canal and nerve damage is essential to the success of these interventions [2,12,13,14]. For this reason, in order to provide an anatomical localization of the mandibular canal, mental nerve, and dental roots, an accurate pre-procedural planning is usually based on high-resolution cone beam computed tomography (CBCT) images [15]. The current strategy to limit nerve damage with freehand incision is to perform a conservative cutting of the cortical plate in the apical portion of the ramus [12,16].

Intra-operative surgical guidance may overcome the limits of freehand surgery, as successfully shown in the computer-guided implant surgery based on stereolithographic printing of templates able to guide drilling and implant insertion [17,18,19,20,21,22]. Computer guided bone harvesting may in fact improve the safety of the procedure as well as reduce variability due to skill factors, thus improving the technical predictability of the intervention. Fully digital workflows were explored to support horizontal ridge augmentation with intraoral bone blocks [23]. Computer Assisted Surgery (CAS) based on computer guided bone harvesting has been proposed to guide the in situ autogenous onlay grafting technique to augment horizontal bone defects of the anterior maxilla [24].

Similarly to those technologies, a computer guided approach to harvest the bone from the mandible has been recently presented [25,26]. By this novel approach, a stereolithographic template is designed during the pre-procedural surgical planning on CBCT 3D images and fitted to the harvesting site cortical bone to guide the movement of the cutting tool [27]. In this way, the position, angulation, and depth of the osteotomy is controlled, optimizing the volume of the harvestable bone block while reducing the risk of damage to anatomical structures close to the donor site. The osteotomy lines, in terms of length, direction and depth, are planned in advance using dedicated software, and the case-specific stereolithographic surgical guide is generated through a computer aided design/computer aided manufacturing (CAD/CAM) process [27]. The first published case-report [25] showed that this approach allowed the surgeon to perform the planned osteotomy lines in accordance with the surgical plan with a minimal discrepancy, and an adequate bone volume was effectively collected in relation to the alveolar defect, suggesting that the technique was clinically feasible and safe, as further confirmed by a subsequent short case series [26]. However, to be proposed as a safe and effective procedure, the typical spatial and angular accuracy achievable with this technique should be evaluated and quantified by using relevant metrics and defining clinically relevant indications. A comprehensive framework for accuracy validation has recently been proposed to systematically and automatically quantify the positional and angular errors between planned and real osteotomy planes [28]. A set of metrics were validated on both human cadaver heads and patients who underwent autogenous bone grafting for dental implant placement and proved effective in quantitatively comparing the real outcome of the procedure with the planned osteotomy geometry [28]. 

The aim of this radiological cone bean computer tomography (CBCT) study was to quantitatively assess the accuracy and technical predictability of this novel computer guided technique that is applied to bone harvesting from the external oblique ridge. The assessment was performed in a cohort of consecutively treated patients providing evidence-based safety margins to be considered during planning. Accordingly, the primary endpoint of the study was the quantification of the spatial and angular displacements between planned and actual cuts obtained with the patient-specific stereolithographic surgical guide generated through a CAD/CAM process. 

Furthermore, this study investigated the impact, in terms of accuracy, of three different strategies available to position the cutting guide at the donor site. Specifically, the following null hypothesis was challenged: “no difference in accuracy is present whether the antero posterior (i.e., mesial-distal) placement of the surgical guide is obtained exploiting one of the following strategies: using CBCT bone surface data; using bone surface data and a built-in reference point to the closest tooth obtained from CBCT data; using bone surface data and a built-in reference point to the closest tooth obtained from both CBCT and intraoral scan data”.

## 2. Materials and Methods

### 2.1. Study Population and Patients’ Groups

The study had an observational retrospective design. Twenty-two consecutive subjects were considered among partially edentulous patients, presenting deficient bone quantity for implant placement and who were treated for an autogenous bone augmentation procedure according to the protocol of De Stavola et al. [25,26,27] by a single surgeon who also developed the planning and designed the surgical guide. All patients were fully informed about the surgical procedures and treatment alternatives and agreed to the proposed treatment at the time of surgery. Indications for treatment were the presence of a severe bone atrophy of the alveolar ridge in the horizontal and/or vertical plane, and a sufficient bone quantity in the donor area of the mandible (external oblique ridge and/or ramus). Treatment exclusion criteria consisted of bone defects following tumor resection, heavy smoking habits (more than 10 cigarettes per day), severe renal and liver disease, a history of radiotherapy in the head and neck region, chemotherapy for treatment of malignant tumors at the time of the surgical procedures, uncontrolled diabetes, active periodontal disease involving the residual dentition, mucosal disease in the areas to be treated, poor oral hygiene, and non-compliance with autogenous bone augmentation surgery. 

According to patients’ clinical characteristics and situation, the surgical guide was planned, exploiting different ways to set the guide in position before the surgery. More specifically, each included subject was treated implementing one of the following three different strategies to allow the mesial-distal guide alignment: the surgical guide was planned using only data of bone surface (group A, seven patients); the surgical guide was planned using only CBCT data of the bone and teeth surface, and realized with a built-in reference point to the closest tooth (group B, eight patients); and the surgical guide was planned using data of both bone (CBCT data) and teeth surface (from both CBCT and intra-oral scan data), and realized with a built-in reference point to the closest tooth (group C, seven patients). 

The observational retrospective design did not require the approval of an ethics committee, as per Italian legislation on clinical investigations at the time of the study. Nevertheless, the investigation was carried out following the rules of the Declaration of Helsinki of 1975, revised in 2013, and performed according to the principles of the ICH Good Clinical Practice.

### 2.2. Computer-Aided Surgical Planning

Pre-operative analysis included a complete medical history, a clinical and radiological examination of the stomatognathic system, and a thorough analysis of the implant recipient site as well as of the bone donor site. Cross sectional images using CBCT (Newtom Giano, Cefla, Charlotte, NC, USA) were obtained preoperatively, with isotropic image reconstruction of 0.3 mm and a field of view of 8 × 11 cm, for assessing the crest dimension and for planning the bone block harvesting. 3D digital dental impressions were also acquired for the patients of group C using an intraoral scanner (Trios 3, 3Shape, Copenhagen Denmark). The planning protocol followed the procedure described in the International Patent N. PCT/IB2014/061624 [27]. Briefly, four steps were realized as described below. The CBCT Digital Imaging and Communication in Medicine (DICOM) datasets were processed with a diagnostic and analysis software (RealGUIDE™ Software Suite, 3Diemme, Cantù, CO, Italy) and the mesial-distal linear defect dimension was measured. Ideal bone cutting planes were defined through each cross-sectional image, keeping a minimum safety margin of 1 mm from the anatomical structures to preserve, such as roots and mandibular canal. Once the cutting planes were established, their projections outside the bone surface were used to define the internal facets of the surgical guide. Each facet was thought to guide the cutting tool direction once this was lent against the surface of the surgical guide. The final guide design was shaped making use of CAD software and then produced in medical polyamide through a CAM process. In those cases where a STereo Lithography interface format (STL) file of the residual dentition was available (from the original DICOM data or from and intraoral scan), a built-in reference point to the closest tooth was designed to facilitate the mesial-distal alignment of the guide.

### 2.3. Surgical Procedure

**Figure 1:**
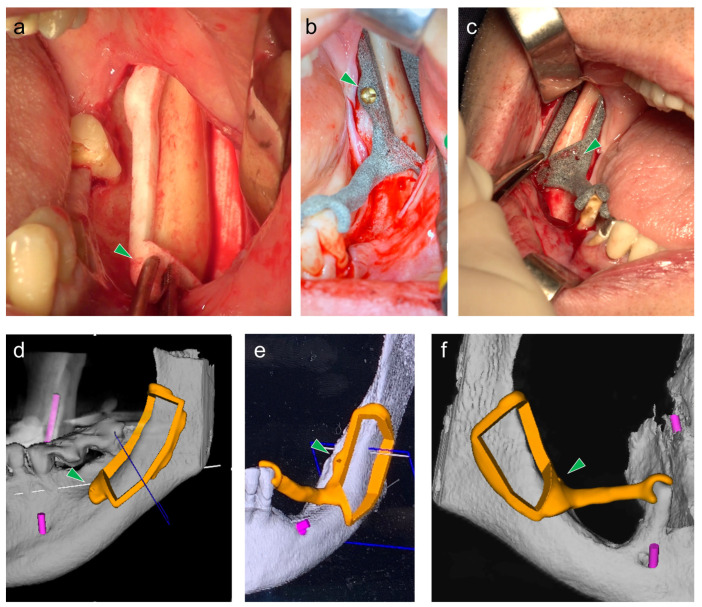
Representative pictures of the surgical fields (top row) and corresponding 3D renderings of the surgical guides (bottom row) for each study group. The surgical guide was designed and positioned according to one of the three following strategies: (**a**,**d**), group (A) using CBCT bone surface data; (**b**,**e**), group (B) using bone surface data and a built-in reference point to the closest tooth obtained from CBCT data; (**c**,**f**), group (C) using bone surface data and a built-in reference point to the closest tooth obtained from both CBCT and intraoral scan data. Before bone incision, the surgical guide was securely stabilized to the bone by placing one 1.3 mm-diameter screw in the built-in hole of the surgical guide (green arrowhead).

The surgical interventions were performed according to the protocol described in De Stavola et al. [25,26], and is briefly summarized here. A full thickness flap was elevated evidencing the external oblique ridge and the lateral aspect of the ramus as well as the lateral aspect of the mandibular body. The surgical guide was placed in the donor site, finding its planned position by checking the best fit between the bone surface and the guide shape. When available (group B and C), the tooth-reference point was also exploited to set the guide in position by simply leaning the guide extension on the pre-defined tooth reference point. No mechanical support was obtained by the tooth. In all cases, the surgical guide was securely stabilized to the bone by placing one 1.3 mm-diameter screw in the built-in hole of the surgical guide. Representative pictures of the surgical fields and corresponding 3D renderings of the surgical guides for each study group are shown in Figure 1.

The osteotomy cuts were made facing the flat side of the piezoelectric insert to the internal face of the surgical guide. The cutting direction was unequivocally defined by the surgical guide, while the working depth was defined by the volumetric image analysis. The block was then removed by a straight thin elevator without the necessity of hammering. The flap was sutured with single and/or mattress sutures and the bone block was then grafted at the defect site following Khoury’s bone augmentation approach [11].

Post-operative CBCT data were acquired, applying the same acquisition settings of the pre-operative scans as a standard protocol after the bone augmentation procedure to ensure the good result of the bone augmentation surgery and excluding any complications which could require immediate treatment. 

According to the specific treatment plan, implants were placed in the reconstructed alveolar crest after 4 months and were prosthetically loaded after an additional four months of healing time.

### 2.4. Accuracy Assessment

**Figure 2:**
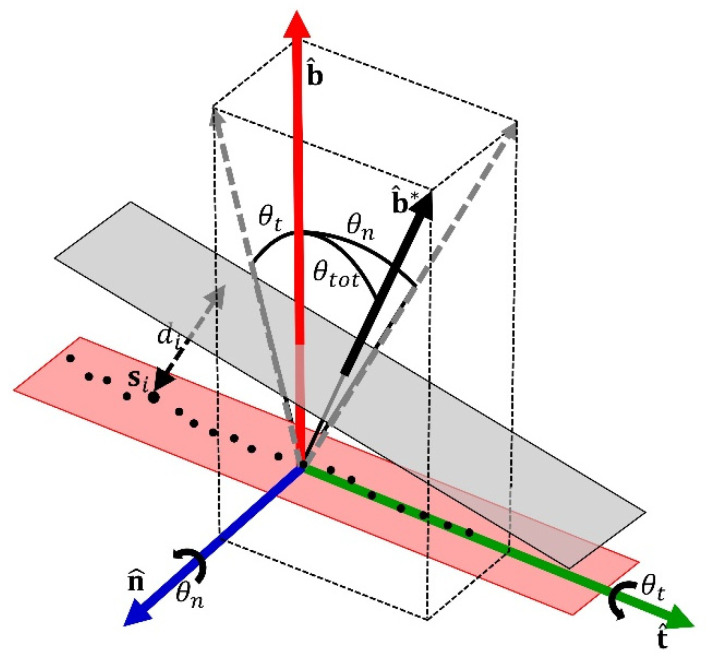
Definition of the six indexes for the quantification of accuracy between the real cut plane (red area) and the planned cut plane (grey area). The three displacement errors, root mean square displacement error (*δ*_RMS_), signed average displacement (*δ*_mean_), residual standard deviation (*δ*_b_), were computed from the distances $d_{i}$ of points $s_{i}$ on the incision surface from the ideal cut plane. The three angular discrepancies were obtained from the direction of the normal to the ideal cut plane ${\hat{b}}^{*}$ in relation to three orthogonal axes defined on the real cut plane: $\hat{b}$, normal to the real cut plane; $\hat{n}$, normal to the cut direction; and $\hat{t}$, tangent to the cut direction. The angle between ${\hat{b}}^{*}$ and $\hat{b}$ is the overall angular error (θ_tot_), which is divided in two components: the signed around-tangent angular error (θ_t_), and the around-normal angular error (θ_n_). Further details are reported in [28].

The pre-operative CBCT data of the donor site were compared to the immediate post-operative CBCT data. The accuracy analysis and the accuracy indexes were defined according to the procedure described by Cristoforetti et al. [28]. Briefly, a rigid registration between pre- and post-surgical mandibular models was automatically accomplished by minimizing the sum of squared distances via a stochastic multi-trial iterative closest point algorithm. Bone harvesting accuracy was quantified by calculating a full set of angular and displacement indexes between the planned and the real cutting planes which characterized the block harvesting procedure. Each block was composed of a variable number of incisions, ranging from four to six according to the local anatomy and bone availability. Incisions were undertaken on four main cut positions: the mesial, distal, medial, and apical. The quantitative accuracy assessment was performed on each incision by automatically measuring the angular discrepancies and the linear displacement between the points on the bone cut and the planned facets of the virtual guide (Figure 2). 

A set of discrepancy metrics (indexes) was defined according to parameters validated previously [28]. The metrics covered different aspects of clinical significance, were tailored to the specific problematics of the osteotomy, and are briefly detailed below, distinguishing between displacement and angular discrepancies. 

Displacement accuracy was assessed measuring three indexes. The *root mean square displacement error* (*δ*_RMS_) represented an overall deviation from the optimal incision surface. The *signed average displacement* (*δ*_mean_), was used to discriminate between a conservative resection (positive value), having no adverse effects apart from harvesting a minor graft volume and an excessive bone removal (negative value), which may have critical consequences on the outcome. The *residual standard deviation* (*δ*_b_) was considered as a quality parameter for plane fitting of the incision surface, thus constituting a detector of deviations from cut flatness and a measure of piezoelectric tool irregularity along the cut.

The angular accuracy assessment was evaluated by computing the angular discrepancy between the planned and effective incision planes, separated into rotational components around normal and tangent axes. Three angular accuracy indexes were defined. The *overall angular error* (θ_tot_) was calculated between the planned and the effective cut plane. The *signed around-tangent angular error* (θ_t_) indicated the angulation of the piezoelectric tool compared to the planned cutting plane, whose positive or negative values were indicative of a conservative cut or an excessive removal of cancellous bone deep into the block. Finally, the *around-normal angular error* (θ_n_) characterized the obliquity of the incisions in the mesial-distal or cranial-apical directions and indicated a difference in the resected material from one cut end to the other, complementing the safety information provided by the signed average displacement.

The confidence of the accuracy indicators was also estimated according to the method by Cristoforetti et al. [28]. A dominant component of uncertainty was derived from the finite resolution of the native images (0.3 mm) and its propagation in the segmentation and registration algorithm. The RMS of the residual distances between pre and post aligned mandibular models amounted to less than 0.4 mm for all the cases, while the alignment optimization algorithm showed a high repeatability with a variability < 0.01 mm. A minor contribution to uncertainty arose from the fitting of the effective cut planes on the cut surfaces of the registered models. The corresponding standard errors were computed for the six discrepancy indexes.

### 2.5. Statistical Analysis

Values obtained for each of the six accuracy indexes were analyzed over the whole study population and according to the four main cut positions: mesial, distal, medial, and apical. Statistical descriptors of accuracy indexes were also calculated for each patient group.

A Shapiro-Wilk test was used to check for the normality of data distribution. Non-normally distributed data were expressed by the median, first quartile, and third quartile of their distributions. A Kruskal-Wallis non-parametric test was applied to identify significant differences among the four cut positions and among the three patients’ groups. A Mann-Whitney U-test was performed between group pairs. 

The dispersion of accuracy data among different patients’ groups was considered as an indicator for the predictability of the procedure. The non-parametric Ansari-Bradley test was used to test statistical differences in the dispersions of accuracy data among the different cut planes and among the three patient groups after subtracting the median of the data distribution. Bonferroni post hoc correction was applied for multiple comparisons. A *p*-value <0.01 was considered statistically significant. The statistical analysis was performed using the MATLAB platform (The MathWorks, Inc. Natick, MA, USA).

## 3. Results

**Figure 3:**
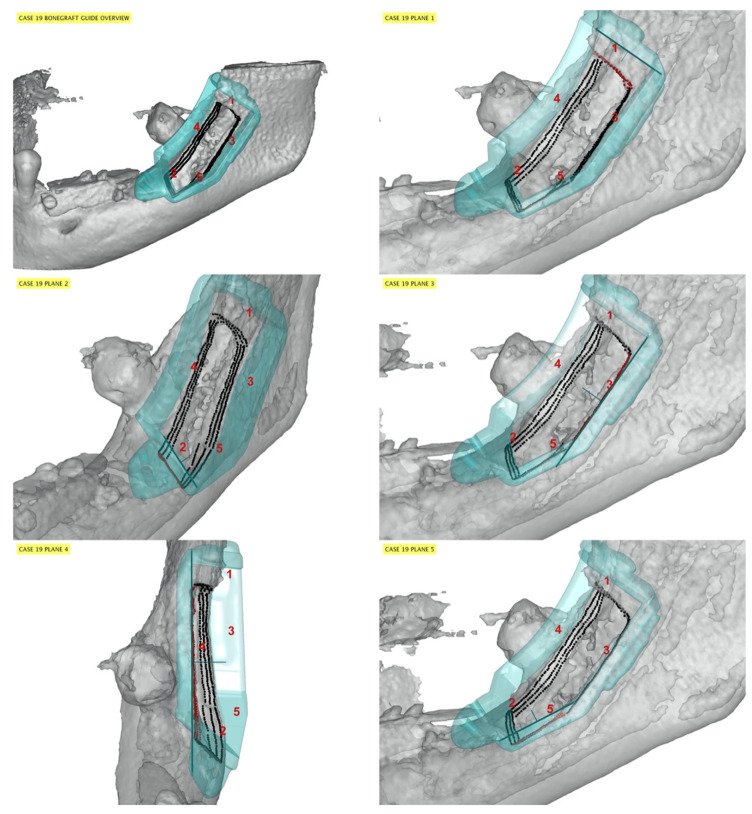
Representative case from group A. The guide model (cyan) and its planes (thick cyan lines) are viewed from the parallel direction. The cut samples (black points, red when corresponding to a specific guide plane) are placed on the post CT soft bone (grey). Due to the lack of a position reference point, the surgical guide was fixed, during the surgery, in a more mesial position compared to the planned position. Red numbers indicate osteotomy cuts: mesial (plane 2) and distal (plane 1) osteotomies were more negatively affected in terms of accuracy compared to the medial (plane 4) and apical ones (planes 3 and 5).

**Figure 4:**
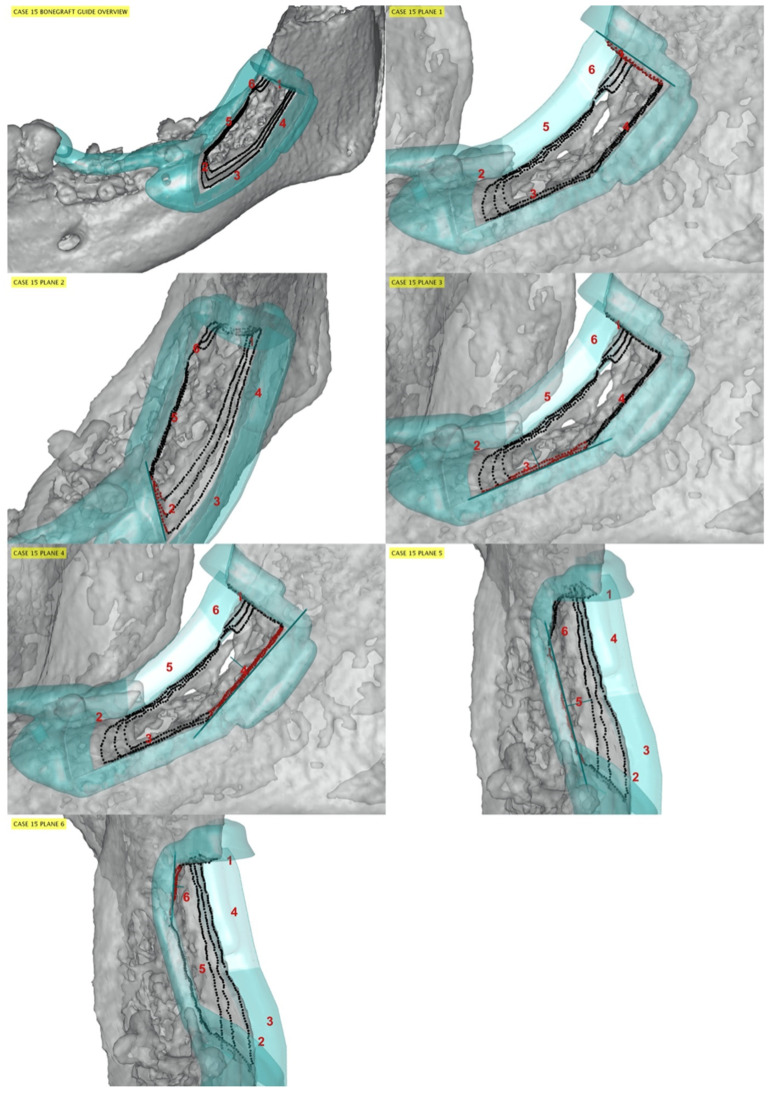
Representative case from group B. The guide model (cyan) and its planes (thick cyan lines) are viewed from a parallel direction. Red numbers indicate osteotomy cuts: plane 1: distal; plane 2: mesial; plane 3–4: apical; plane 5–6: medial. The cut samples (black points, red when corresponding to a specific guide plane) are placed on the post CT soft bone (grey). The surgical guide was minimally shifted mesially compared to the planned position.

**Figure 5:**
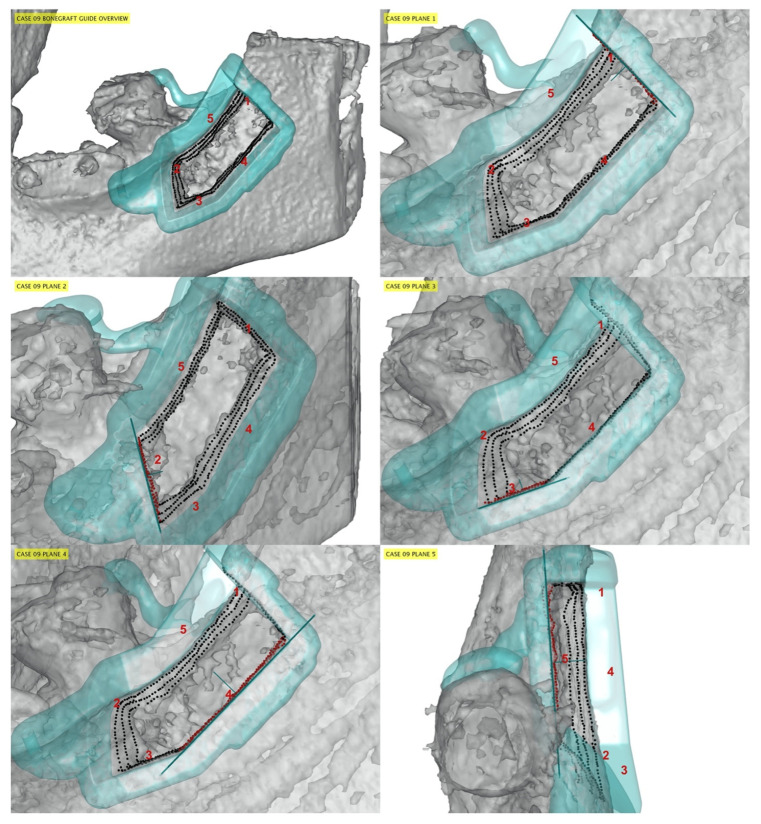
Representative case from group C. The guide model (cyan) and its planes (thick cyan lines) are viewed from a parallel direction. Red numbers indicate osteotomy cuts: plane 1: distal; plane 2: mesial; plane 3–4: apical; plane 5–6: medial. The cut samples (black points, red when corresponding to a specific guide plane) are placed on the post CT soft bone (grey). Note the extreme accuracy between planning and execution of the osteotomy lines.

One-hundred-and-twenty bone osteotomy cuts were surgically performed, and 119 cutting planes were included in the accuracy analysis. The distal bone osteotomy from one patient (group A) was not evaluated because the cutting plane was incidentally outside the field of view of the post-operative CBCT image. Representative cases are presented in Figure 3, Figure 4 and Figure 5 for patients from groups A, B, and C, respectively. 

### 3.1. Overall Accuracy and Cutting Planes Features

**Figure 6:**
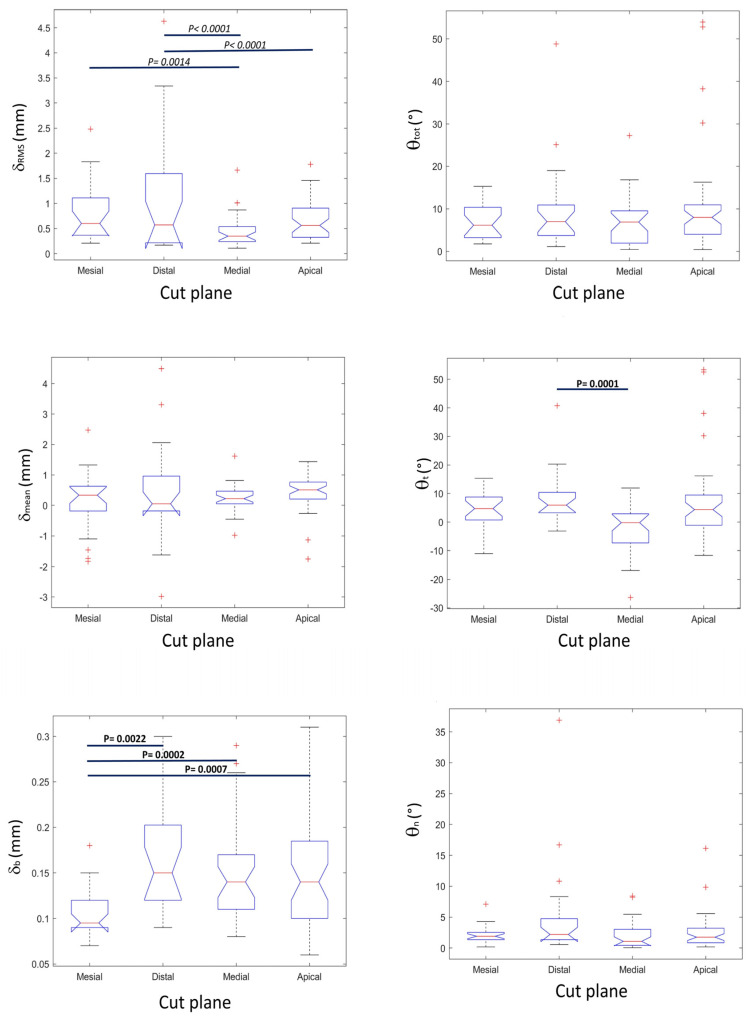
Box and whiskers plots of the six accuracy indicators computed on the four osteotomy cut directions: mesial, distal, medial, apical. From top to bottom and from left to right: *root mean square displacement error* (*δ*_RMS_), *overall angular error* (θ_tot_), *signed average displacement* (*δ*_mean_), *signed around-tangent angular error* (θ_t_), *residual standard deviation* (*δ*_b_), and *around-normal angular error* (θ_n_). Red crosses indicate outliers. Horizontal bars connect groups of data having significantly different distributions: *p*-values in bold represent significance to Mann-Whitney test (different medians), while *p*-values in italics represent significance to Ansari-Bradley test (different dispersions). Reported *p*-values include a Bonferroni multiple comparison post-hoc correction.

Results of accuracy indexes over the whole study population, broken down by cutting facet positions, are presented in Figure 6, and the main statistical descriptors are summarized in Table 1. The median (first quartile-third quartile) values over all the facets in the whole study population for *δ*_RMS_, *δ*_mean_, and *δ*_b_ were 0.52 (0.30–0.97) mm, 0.28 (0.05–0.62) mm, and 0.14 (0.10–0.17) mm, respectively. Angular discrepancy indexes showed median (first quartile-third quartile) values for θ_tot_, θ_t_, and θ_n_ of 6.91 (3.30–10.12)°, 3.29 (−1.11–8.79)°, 1.77 (0.90–3.22)°, respectively. The confidence intervals of the displacement indexes resulting from plane fitting were <0.1 mm, inferior to data resolution and registration error (0.4 mm), while those of the angular discrepancy indexes were < 2° for most of the examined cases.

Considering that negative values for *δ*_mean_ and θ_t_ are associated with the removal of a higher amount of bone than planned, the results document the safety of the procedure, guaranteeing the safeguard of the sensitive anatomical structures. The analysis of accuracy data broken down according to cutting plane (Table 1 and Figure 6) showed that the majority of the cuts were conservative, with *δ*_mean_ > 0 and θ_t_ > 0. The lowest θ_t_ value was −11.6°. In the medial and apical cuts, which are most critical because they are closer to sensitive anatomical structures, *δ*_mean_ ranged from −1.75 mm to 1.29 mm, exceeding the 1 mm safety margin in only two out of 64 (3.1%) osteotomies.

The statistical comparison of angular and displacement discrepancies among the four main cutting planes demonstrated that the *δ*_b_ values of mesial osteotomies were significantly lower than those of medial, distal, and apical cuts. In addition, θ_t_ values of distal osteotomies were significantly higher than those of medial cuts. No other statistically significant differences were noted among the different cutting planes for the other discrepancy indexes.

A higher predictability was associated to medial and apical cuts. More specifically, *δ*_RMS_ values of medial cuts were statistically less disperse than *δ*_RMS_ values of distal and mesial cuts. The same apply for the *δ*_RMS_ values of apical cuts with respect to distal cuts. No significant differences were present in the dispersions of the other accuracy indexes among the different cutting planes.

### 3.2. Groups Analysis

**Figure 7:**
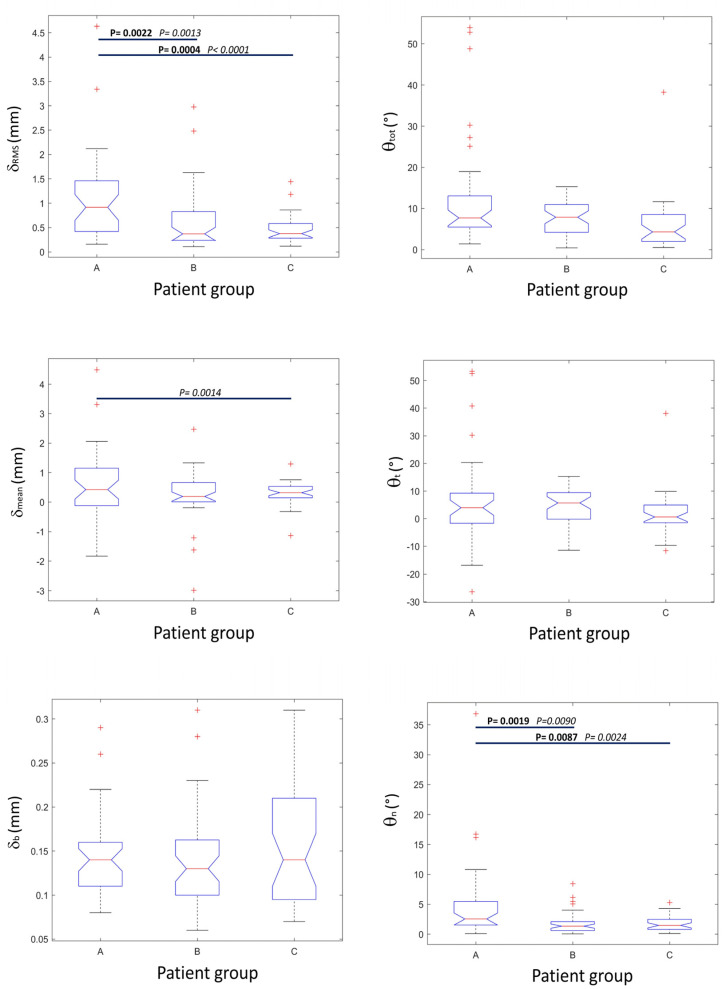
Box and whiskers plots of the six accuracy indicator values grouped according to patient group. From top to bottom and from left to right: *root mean square displacement error* (*δ*_RMS_), *overall angular error* (θ_tot_), *signed average displacement* (*δ*_mean_), *signed around-tangent angular error* (θ_t_), *residual standard deviation* (*δ*_b_), and *around-normal angular error* (θ_n_). Red crosses indicate outliers. Horizontal bars connect groups of data having significantly different distributions: *p*-values in bold represent significance to the Mann-Whitney test (different medians), while *p*-values in italics represent significance to the Ansari-Bradley test (different dispersions). Reported *p*-values include a Bonferroni multiple comparison post-hoc correction.

Accuracy results broken down according to patients’ subgroups are shown in Figure 7, and statistical descriptors are summarized in Table 2. Data analysis showed that a lower accuracy was obtained in osteotomies of patients from group A in respect to both groups B and C. Two out of six accuracy indexes, namely *δ*_RMS_ and θ_n_, showed significantly higher values in the A group [*δ*_RMS_ = 0.91 (0.43–1.42) mm, θ_n_ = 2.54 (1.56–5.49)°] than in group B [(*δ*_RMS_ = 0.37 (0.24–0.81) mm, θ_n_ = 1.35 (0.61–2.10)°] and in group C [(*δ*_RMS_ = 0.37 (0.29–0.58) mm, θ_n_ = 1.48 (0.8–2.44)°]. No statistical difference for any of the accuracy indexes was noted between group B and C.

Groups B and C also resulted in a higher predictability of the osteotomy cuts with respect to group A. More specifically, the values of *δ*_RMS_ and θ_n_ were statistically more disperse in group A than in group B and C. The values of *δ*_mean_ were also statistically more dispersed in group A than in group C. No significant differences were present in dispersions of accuracy indexes between group B and C.

## 4. Discussion

Autogenous bone harvesting is a skill dependent procedure. Although in the literature a minimal risk has been associated to this surgical procedure [16,29], the possibility of major complications should be considered. Guided bone harvesting with CAS may improve the safety of the procedure as well as reduce variability due to skill factors, thus improving the technical predictability of the intervention. Findings of this study showed that accuracy values of *δ*_RMS_ equal to 0.52 (0.30–0.97) mm and θ_tot_ equal to 6.91 (3.30–10.12)° were associated with the CAS procedure proposed by De Stavola et al. [25,27]. These results were further improved when the closest tooth was used as a reference landmark to optimize the mesial-distal alignment of the guide. Values of *δ*_RMS_ equal to 0.37 (0.28–0.58) mm and θ_tot_ equal to 4.30 (2.00–8.55)° were achieved when data from CBCT and an intraoral scan were considered in the design the surgical guide. 

In the literature, several CAS approaches were presented in the field of mandibular reconstruction, with a variety of implementations and different reported deviations, ranging from 0–12.5 mm for displacements and 0.9–17.5° for angles [30]. One approach of Virtual Planning Surgery (VPS) with a 3D printed guide showed deviations between 1.76–3.11 mm, with a decreased accuracy for long donor bone segments [31]. Other mandibular VPS accuracy studies, on a smaller numbers of patients, reported deviations of 2.00 mm and 1.30 mm for mandibular and fibular osteotomies [32], 0.2–2.7 mm [33], 2.06 mm [34], and 0.9–1.3 mm for fibular size [35], 1.8 mm for resection planes and 3.0 mm and 4.2° for fibular length and angulation [36], 3.06–3.76 mm and 8.5–10.4° for resection planes [37], and of 2.92–5.61 mm and 3.85–5.88° for spatial and angular cutting plane deviations [38]. Worthy of note are recent works reporting on 3D-navigated VPS as an alternative to 3D printed cutting guides for mandibular osteotomy. 3D-navigation was applied to the cutting tool directly [39], obtaining an angular deviation of 8.08°and a spatial deviation of 2.63 mm for the osteotomy trajectory. Optical tracking of the saw [40] reduced median distances from the virtual plane with respect to the unnavigated approach from 1.2 mm to 2.1 mm, and median angles from 3.5° to 4.5° (pitch), and from 2.6° to 7.4° (roll). Another alternative approach was to navigate the surgical guide instead of the saw by electromagnetic tracking [41], achieving a spatial accuracy of 1.1 mm and angular deviations of 1.8° (yaw) and 1.6° (roll), potentially offering the same accuracy of 3D-printing with reduced costs and treatment time.

While this accuracy range can be adequate in the contest of mandibular reconstruction, in the case of intraoral bone grafting in the ramus for autogenous bone augmentation, a greater focus is required to avoid interference with nearby anatomical structures and to reducing the risk of neurological damage. A CAS method has been recently exploited to guide an in-situ grafting technique to augment horizontal bone defects of the anterior maxilla [29], showing a promising clinical outcome. Fully digital workflows are under investigation to support horizontal ridge augmentation by CAS intraoral bone block grafting. An in vivo study validated the approach showing root mean square (RMS) deviations of 0.37 mm, inferior to those with conventional surgery (0.72 mm) [28], while an in vitro study on 3D printed models reported an accuracy of 0.1–1.7 mm [42].

In the procedure proposed by De Stavola and co-workers [25,27], the osteotomy lines can be planned in advance, thus optimizing the balance between the quantity of the harvested bone and the specificity of each patient’s anatomy. The accuracy of the procedure is essential to properly understand its safety, reproducibility, and technical predictability. The achieved *δ*_RMS_ discrepancy value of 0.52 (0.30–0.97) mm is compatible with a safe surgical procedure when associated with the indication of maintaining a safety margin of 1 mm from delicate anatomical structures. A further reduction of the displacement error may be too complex to be achieved, considering the typical spatial resolution of the CBCT images used during planning and taking into account that the piezoelectric tools have a thickness ranging from 0.5 mm to 0.6 mm. 

The osteotomy lines planned in the treated cases that are representative of a typical bone harvesting procedure were limited in depth, ranging from 3 mm to 8 mm. In these conditions, although angular displacements were present, corresponding linear displacements from the planned cutting planes were intrinsically limited. A larger safety margin should be considered in case the osteotomies are to be performed deeper into the bone. 

Different accuracy values were obtained according to the cutting planes, depending mainly on site accessibility and local bone characteristics. The largest displacement discrepancies occurred in the mesial and distal osteotomies due to the inaccurate positioning of the surgical guide, resulting in a moderate shift in the antero-posterior direction. However, the risk associated to the inaccurate position of these osteotomies is limited, usually being far from sensitive anatomical structures. In contrast, both signed discrepancies *δ*_mean_ and θ_t_ were particularly important for the apical and medial cuts, where excessive bone harvesting may increase the risk of nerve damage. It is noteworthy that all of the possible discrepancy contributions described above (shift, inclination, and non-planarity of the cut), affect the *δ*_RMS_, which, in fact, could be considered the main overall discrepancy index. 

The accuracy analysis among the three patients’ groups, corresponding to different methods of planning and making the surgical guide, showed that planning the surgical guide using both bone CBCT data and a position reference point, based on the STL or DICOM file of a tooth, (groups B and C), guarantees a more accurate surgical guide placement than using bone CBCT data alone (group A). Study data therefore support the rejection of the null hypothesis stated in the introduction section. Indeed, the absence of a tooth-related reference point reduced the overall accuracy of the intra-surgical guide position and produced the largest displacements between planned and real positions in the anterior-posterior (i.e., mesial-distal) direction. Statistical differences on relevant accuracy indexes were found for both spatial and angular data, indicating that a surgical guide including a tooth-related reference point should be preferred when planning and executing a bone harvesting surgery using a stereolithographic guide. However, it should be noted that a shift of the surgical guide position in the anterior-posterior direction minimally affects the positions of the corresponding medial-vertical cuts and apical osteotomies. This point is surgically relevant since medial and apical incisions are closer to the alveolar canal, posing a higher risk of damage for sensitive anatomical structures. Although the safety of the procedure is minimally affected by a moderate displacement of the guide in the anterior-posterior direction, the collection of both DICOM and intra-oral STL files is strongly recommended for procedure planning in order to optimize the surgical performance in terms of accuracy and patient safety.

This study has some limitations. All cases included in this series were treated by a single surgeon who also performed the planning and designed the surgical guide. Further studies, possibly having a prospective design, are required to address variability due to different surgeons. In fact, different results, in terms of accuracy, cannot be excluded for other surgeons following the same procedure reported here. However, the specific design of the surgical guide allows for a conservative bone resection, but minimizes the risk of collecting more bone than planned. Thus, this CAS approach, combined with a smart design of the surgical guide, properly implements safety-by-design principles [43]. 

Another limitation is the confidence associated with the accuracy measurements. The accuracy data of most of the studies presented in the literature reporting accuracy data were obtained comparing pre- and post-surgical images using manual measurements [32,33,34,37], which are affected by intra and inter observer variability [37], and consequently required repeated multi-observer quantification. We were able to assess the accuracy of the procedure with a previously validated method [26] which was based on an automatic algorithm that was not affected by variability with respect to repetition or by human bias, and limited in accuracy only by the resolution and quality of the CBCT input images. Changes in the resolution of CBCT and intraoral scan data could be associated with the modification of the accuracy of the procedure [28].

A further potential source of variability could be associated with the stability of the reference tooth when the surgical guide includes a fitting extension similar to the one realized in groups B and C. However, in this study, no specific requirements were set in terms of stability for the reference tooth, considering that no mechanical load was applied to the tooth and an effective fixation of the surgical guide to the harvesting site was obtained by applying a bone screw. 

Finally, a decrease in accuracy associated to the osteotomy length cannot be excluded. The findings of this study showed similar accuracy values independently from the block dimension, but the accessibility of the harvesting site and the position of the single cut could have an impact, thus indirectly affecting the accuracy of long cuts extending over difficult-to-access anatomical districts.

## 5. Conclusions

Computer-guided bone harvesting, performed according to the protocol described by De Stavola and colleagues [25], effectively translated the surgical plan into the surgical field, assisting the surgeon in performing the correct osteotomy and limiting the variability of the cut position due to skill factors. A safety margin of 1 mm should be considered between planned osteotomies and any anatomical structure to be preserved.

The results of this study are relevant for both clinicians and patients. Technical difficulties and the risk of a freehand approach limit the current implementation of bone harvesting procedures in the retromolar area, thus precluding the accessibility of some therapeutic options that could be offered to the patient needing important bone augmentation before dental implant placement. The learning curve for performing bone harvesting procedures in the retromolar region using a surgical guide could be shorter than that for a hands-free surgical intervention, improving reproducibility among different surgeons and providing higher confidence for younger surgical practitioners.

## Figures and Tables

**Table 1:** Results of accuracy indexes over the whole study population, broken down by cutting facet positions.

	Displacement Indexes (mm) Median (First Quartile–Third Quartile)			Angular Discrepancy Indexes (°) Median (First Quartile–Third Quartile)		
	*δ*_RMS_	*δ*_mean_	*δ*_b_	θ_tot_	θ_t_	θ_n_
Mesial osteotomy	0.60 (0.37–1.11)	0.33 (−0.18–0.63)	0.095 (0.09–0.12)	6.12 (3.23–10.37)	4.73 (0.74–8.73)	1.91 (1.35–2.54)
Distal osteotomy	0.57 (0.22–1.59)	0.05 (−0.18–0.96)	0.15 (0.12–0.20)	6.98 (3.75–10.91)	5.89 (3.26–10.38)	2.20 (1.35–4.76)
Medial osteotomy	0.35 (0.24–0.54)	0.22 (0.05–0.46)	0.14 (0.11–0.17)	6.88 (1.95–9.53)	−0.20 (−7.31–2.91)	1.08 (0.43–3.03)
Apical osteotomy	0.56 (0.33–0.91)	0.51 (0.21–0.77)	0.14 (0.10–0.18)	7.98 (4.06–10.95)	4.39 (−1.10–9.43)	1.76 (0.85–3.21)
Overall	0.52 (0.30–0.97)	0.28 (0.05–0.62)	0.14 (0.10–0.17)	6.91 (3.30–10.12)	3.29 (−1.11–8.79)	1.77 (0.90–3.22)

**Table 2:** Results of accuracy indexes broken down according to study subgroups.

	Displacement Indexes (mm) Median (First Quartile–Third Quartile)			Angular Discrepancy Indexes (°) Median (First Quartile–Third Quartile)		
	*δ*_RMS_	*δ*_mean_	*δ*_b_	θ_tot_	θ_t_	θ_n_
Group A	0.91 (0.42–1.46)	0.42 (−0.12–1.15)	0.14 (0.11–0.16)	7.72 (5.49–13.06)	3.97 (−1.68–9.23)	2.54 (1.54–5.49)
Group B	0.37 (0.24–0.83)	0.19 (0.01–0.66)	0.13 (0.10–0.16)	7.90 (4.21–10.95)	5.72 (−0.18–9.43)	1.35 (0.60–2.11)
Group C	0.37 (0.28–0.58)	0.32 (0.14–0.53)	0.14 (0.09–0.21)	4.30 (2.00–8.55)	0.60 (−1.50–4.96)	1.48 (0.80–2.47)

## Data Availability

All data generated or analyzed during this study are included in this article.
